# Assessment of the Association Between Neuraxial Anesthesia and Back Pain After Delivery: A Systematic Review and Meta-Analysis

**DOI:** 10.1155/anrp/2105413

**Published:** 2025-01-29

**Authors:** Sara Timerga, Getaw Walle, Wondwosen Mebratu, Aynalem Befkadu

**Affiliations:** ^1^Department of Anesthesia, College of Medicine and Health Sciences, Wollo University, Dessie, Ethiopia; ^2^Department of Epidemiology and Biostatistics, School of Public Health, College of Medicine and Health Sciences, Wollo University, Dessie, Ethiopia

**Keywords:** back pain, delivery, epidural anesthesia, meta-analysis, neuraxial anesthesia

## Abstract

**Background:** Back pain after delivery both under cesarean section and spontaneous vaginal delivery is the most common pregnancy-related musculoskeletal problem. There are multiple studies that emphasize the effect of epidural anesthesia and spinal anesthesia on the incidence and severity of postdelivery back pain. There are others stating no association between the two.

**Objective:** The aim of this study is to summarize the relationship between back pain after delivery and neuraxial anesthesia.

**Methods:** Studies identified from database: Cochrane Library, The Virtual Health Library, National Library of Medicine PubMed, Google Scholar, and citation searching with both experimental and observational study design were included. Exposed and nonexposed incidence of back pain was extracted to analyze the pooled odds ratio assessing the association of postpartum back pain and neuraxial anesthesia. Heterogeneity was checked across studies using Cochrane *Q* test statistic and *I*^2^. Small study effect was assessed using a funnel plot graphically and nonparametric rank correlation (Begg) test.

**Results:** Four RCT and 11 observational studies were identified for analysis. The studies included mothers delivering under cesarean section and vaginal delivery with epidural anesthesia, spinal anesthesia, and combined spinal epidural anesthesia. Based on the 15 studies included in this meta-analysis, the pooled odds ratio according to random effect restricted maximum—likelihood model was 1.2 (95% CI (0.77–1.86)) with *p* value = 0.43. There was a significant heterogeneity with *I*^2^ = 97.76%, *H*^2^ = 44.58, and Cochrane *Q* statistics *p* value = 0.001.

**Conclusion:** Our result suggests neuraxial anesthesia may not be the cause or the risk factor for the overwhelmingly high incidence of back pain women experience after delivery.

## 1. Introduction

Back pain after delivery both under cesarean section and spontaneous vaginal delivery is the most common pregnancy-related musculoskeletal problem [1]. According to most studies, at least half of the pregnant population is affected by it [2, 3]. Despite the advancement of medical practice, the etiology of this postpartum back pain is poorly understood.

Postpartum back pain is characterized by a musculoskeletal discomfort at the lower lumbar area that can be caused by a combination of mechanical, hormonal, circulatory, and psychosocial factors [4]. The factors identified to be associated with the incidence of back pain after delivery include neuraxial anesthesia [5–7], previous history of back pain [8], greater weight, younger age [9], and type of neuraxial anesthesia. From these risk factors epidural anesthesia was named as the causative agent by MacArthur, C. and colleagues in 1990 [10] the mechanism they described was postural, related to a combination of stressed positions in labour, muscular relaxation, effective analgesia, and lack of mobility, this was supported by Grove in 1973 [11], Russell et al. in 1993 [8], and Breen et al. in 1994 [9], all of whom agreed epidural anesthesia is main factor associated with incidence and severity of back pain. Since then, there have been studies that have disputed this idea claiming no correlation between back pain and neuraxial anesthesia [12–17].

Over the last 3 decades there have been multiple studies conducted on the effect of neuraxial anesthesia on the incidence of postdelivery back pain. However, the findings have been inconsistent; there are some studies that emphasize the correlation of epidural anesthesia and spinal anesthesia with incidence and severity of postdelivery back pain [10, 18]. And there are other studies that showed no correlation between neuraxial anesthesia and back pain [9, 12, 13].

The purpose of this systematic review and meta-analysis is to summarize literature regarding the relationship between postpartum back pain and neuraxial anesthesia. There is no previous meta-analysis conducted to assess the true relationship of back pain and neuraxial anesthesia in mothers delivering under both cesarean section and vaginal delivery. For studies for which there was sufficient data, we conducted meta-analysis to compile the data from several studies into a single estimate of effect and resolve the inconsistent result seen between studies.

## 2. Methods and Materials

### 2.1. Overview

This study followed a systematic review and meta-analysis recommendation protocol of PRISMA-P [19]. The protocol was registered under the identification number CRD42023484380 in PROSPERO before the start of the review.

### 2.2. Study Eligibility

PECO for searching strategies.

• P-Population: parturient who delivered with and without neuraxial anesthesia
• E-Exposure: delivery with neuraxial anesthesia
• C-Comparison: delivery without neuraxial anesthesia
• O-Outcome: back pain

### 2.3. Inclusion Criteria

Studies that used observational (prospective and retrospective cohorts) and interventional (randomized controlled trials, RCTs) epidemiological designs, comparing the incidence of back pain after delivery with and without neuraxial anesthesia (spinal anesthesia, epidural anesthesia, and combined spinal-epidural anesthesia), were eligible for the study.

### 2.4. Exclusion Criteria

Studies that reported in non-English languages and does not compare the incidence of back pain after delivery and neuraxial anesthesia.

### 2.5. Data Sources

Systematic literature search was conducted from a database: Cochrane Reviews, The Virtual Health Library, National Library of Medicine PubMed, and Google Scholar.

### 2.6. Search Strategy

Search was conducted by author ST and AB starting from May 1^st^ 2024, encompassing studies conducted at any year.

## 3. National Library of Medicine PubMed

By dividing the research question in to concepts and key words “Neuraxial anesthesia”, “back pain”, and “delivery” searching for synonym words we built the following on the search engine.

1. Neuraxial anesthesia
  “Neuraxial anesthesia” OR “Spinal anesthesia” OR “Epidural anesthesia” OR “Epidural analgesia” OR “Combined Spinal-Epidural anesthesia” OR “Caudal anesthesia”
2. Back pain
  “Back pain” OR Backache OR Backaches OR “Post-Dural Puncture Backache”
3. Delivery
  Delivery OR Labor OR Labour OR “Cesarean section”
4. Combined
  ((“Neuraxial anesthesia” OR “Spinal anesthesia” OR “Epidural anesthesia” OR “Epidural analgesia” OR “Combined Spinal-Epidural anesthesia” OR “Caudal anesthesia”) AND (“Back pain” OR Backache OR Backaches OR “Post-Dural Puncture Backache”)) AND (Delivery OR Labor OR Labour OR “Cesarean section”)

### 3.1. The Virtual Health Library

(Neuraxial anesthesia OR spinal anesthesia OR epidural anesthesia) AND (back pain OR backache OR low back pain) AND (labor OR delivery OR caesarean section OR childbirth).

### 3.2. Google Scholar

Back pain childbirth neuraxial spinal and epidural anesthesia “labor” OR “caesarean section” OR “back pain” OR backache OR “spinal anesthesia” OR “epidural anesthesia” OR “epidural analgesia” OR “neuraxial anesthesia” “back pain”.

### 3.3. Cochrane Library

1. MeSH descriptor: [Low Back Pain] explode all trees
2. (back NEXT (pain⁣^∗^ or ache⁣^∗^)): ti, ab, kw AND (low NEXT back NEXT (pain⁣^∗^ or ache⁣^∗^)): ti, ab, kw
3. #1OR#2
4. MeSH descriptor: [Nerve Block] explode all trees
5. (nerve NEXT block): ti, ab, kw AND (spinal NEXT anesthesia): ti, ab, kw AND (epidural NEXT anesthesia): ti, ab, kw AND (combined NEXT spinal NEXT epidural NEXT anesthesia): ti, ab, kw
6. (nerve NEXT block): ti, ab, kw AND (spinal NEXT anesthesia): ti, ab, kw AND (epidural NEXT anesthesia): ti, ab, kw AND (combined NEXT spinal NEXT epidural NEXT anesthesia): ti, ab, kw
7. MeSH descriptor: [Parturition] explode all trees
8. (childbirth): ti, ab, kw AND (Parturition): ti, ab, kw AND (labor): ti, ab, kw AND (delivery): ti, ab, kw
9. #7or#8
10. #3AND#6
11. (childbirth): ti, ab, kw AND (Parturition): ti, ab, kw AND (labor): ti, ab, kw AND (delivery): ti, ab, kw AND (back pain): ti, ab, kw

### 3.4. Study Selection

All citations identified by our search strategy were exported to EndNote, bibliographic management software, and duplicates were removed. The identified articles were read thoroughly by two reviewers SA and AB independently to make sure they are suitable for data extraction. A disagreement between the two reviewers was solved by a third reviewer GW. A hand search was performed on the reference lists of selected articles in order to include studies that were not identified by the search strategy. The search process is presented in a PRISMA flowchart [20].

### 3.5. Data Extraction

Extraction was done using a standardized data extraction format with Microsoft Excel 2010, which was adopted from the Joanna Briggs Institute (JBI) data extraction format, which includes author, publication year, study area, study design, response rate, follow up period, data collection method (retrospective or prospective), region the study was conducted in, sample size, incidence of back pain in exposed, and nonexposed group and the quality score of each study. The extraction form was performed by two independent reviewers SA and AB. Disagreement was fixed through consensus or inviting third neutral reviewer, WM.

### 3.6. Outcome of the Study

The outcome variable was incidence of back pain after delivery. Back pain after delivery is defined by a pain or discomfort localized at the lower back that starts two to 3 hours after neuraxial anesthesia and delivery lasting up to 6 weeks and more [9, 10, 16].

### 3.7. Quality Appraisal

The JBI check list was used to assess the quality of researches based on the listed criteria, and two reviewers assessed for those studies. Based on the quality assessment studies which had seven and more result were identified as qualified to be included in the study.

### 3.8. Data Analysis

Data were analyzed using Stata: Statistical software for data science version 17. Data were presented from eligible studies in evidence table and summaries using descriptive statistics. A forest plot generated to show the individual study effect size with odds ratio (OR) and 95% CI using the random effect Restricted maximum—likelihood model, displaying the author's name, publication year and study weights.

The heterogeneity in the analysis was assessed by the using Cochrane *Q* test statistic with *p* value less < or = 0.1 considered statistical significant heterogeneity and tau^2^ index test expressed as, together with 90% confidence intervals (CI). *I*^2^ was tabulated to assess the inconsistency between studies. For *I*^2^ values of 0%–25% represented minimal inconsistency, 26%–75% represented moderate inconsistency, and values greater than 75% represented substantial inconsistency. Since there was substantial heterogeneity further subgroup analysis was done. In order to detect the outlier studies that resulted in significant heterogeneity Galbraith plot was performed. For small study effect, funnel plot was done with associated Begg's regression tests.

The meta-analyses were performed by calculating ORs from the incidence of postpartum back pain in delivery groups with and without neuraxial anesthesia, extracted from the studies. The random effect restricted maximum—likelihood model was used to assess the effect of neuraxial anesthesia on occurrence of postpartum back pain. Weighted ORs and 95% CI were calculated to pool study and control groups in each publication for analysis.

### 3.9. Subgroup Analysis

Subgroup analysis was conducted to minimize the possible source of heterogeneity by study design (cohort and RCT), data collection (prospective and retrospective), region (Europe, North America, Australia, and Asia), and follow up period (1 year, 6 months, 3 months, and 6 weeks).

## 4. Results

### 4.1. Study Selection

In this systemic review and meta-analysis, a total of 392 articles were identified through search from Cochrane reviews, The Virtual Health Library, National Library of Medicine PubMed, and Google Scholar databases. After removing duplicates, 369 articles remained for further screening. The remaining articles were screened by their title based on which 294 articles were excluded. Abstract of the remaining 75 articles were evaluated for eligibility and, 21 studies were identified for full study evaluation (Supporting file 2). Five of the studies had no comparative group: one compared paramedian approach with median approach for spinal anesthesia without assessing the incidence of back pain in the absence of spinal anesthesia [21], another study assessed the factors associated with the incidence of back pain after delivery with epidural anesthesia [22], one was a study comparing spinal anesthesia performed versus 22-gauge and 25-gauge whitacre needles with 26-gauge quincke needles [23], another was comparison of 26-gauge Atraucan and 26-gauge Quincke Spinal Needles [24] and the last one assessed the prevalence of back pain in mothers who delivered under epidural and spinal anesthesia [25]. Two of the articles were excluded for short duration of follow up period 14 days [7] and 24 h [26], which were not adequate to assess persistent back pain. The last study excluded was a randomized control study that did not meet the required quality to be included [27].

**Figure 1:**
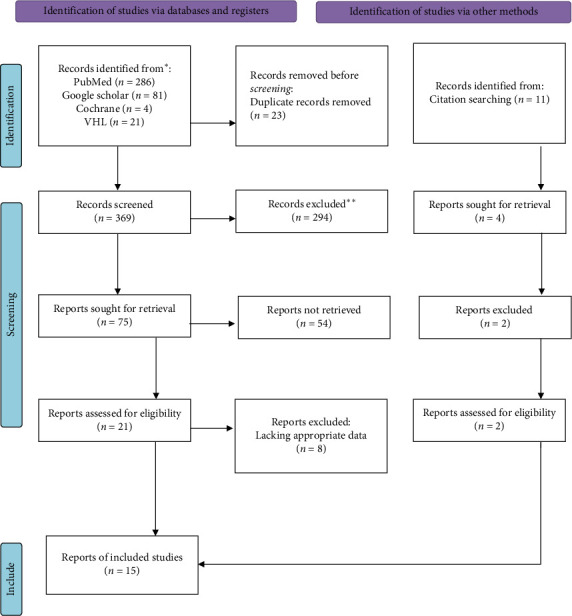
PRISMA flowchart showing search strategies.

From literature citation search, 11 studies were identified, after review 2 studies met the inclusion criteria and were included in the study. In the end, 15 studies were identified to be included in the final analysis Figure 1. The quality assessment showed that the studies were medium and high quality (Supporting file 1).

### 4.2. Characteristics of Included Studies

In this systematic review and meta-analysis, a total of 57711 parturient were included from the 15 studies selected, with sample size ranging from 198 to 40,057. Four of the studies were randomized controlled trials and 11 were cohort studies with both retrospective and prospective data collection methods. The publication dates of the studies were between 1990 and 2022.

Two of the studies were conducted in Asia (Pakistan and Taiwan), two of the studies were from North America (Canada and USA), one was conducted in Australia, and 10 of the studies were conducted in Europe (Turkey, England, Scotland, Sweden, and Lithuania) Table 1.

### 4.3. Meta-Analysis

**Figure 2:**
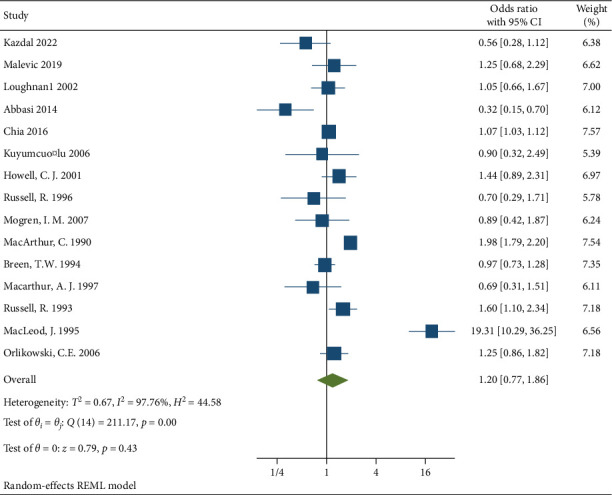
Forest plot on the associations between neuraxial anesthesia and back pain after delivery in all 15 studies.

15810 participants who delivered under neuraxial anesthesia developed back pain. The incidence of back pain after delivery in the neuraxial group of the included studies ranges from 3.9% [14] to 33.1% [18]. Based on the 15 studies included in this meta-analysis, the pooled OR according to random effect Restricted maximum—likelihood model was 1.2 (95% CI (0.77–1.86)) with *p* value = 0.43 Figure 2. There was a significant heterogeneity with *I*^2^ = 97.76%, *H*^2^ = 44.58, and *Q* statistics *p* value = 0.001 Figure 2.

**Figure 3:**
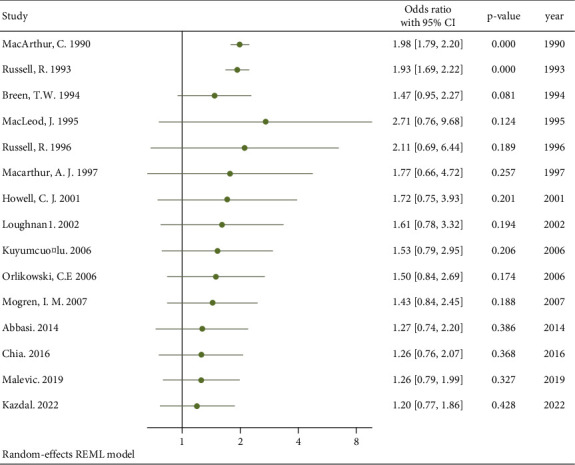
Cumulative meta-analysis based on publication year.

A cumulative meta-analysis of the studies based on the year of publication was conducted to assess the pattern of relationship between postpartum back pain and neuraxial anesthesia over time Figure 3. Over the years, the relationship between neuraxial anesthesia and postpartum back pain has shifted from statistically significant relationship to no association.

### 4.4. Assessing Heterogeneity and Publication Bias

The studies showed significant heterogeneity and inconsistency using Cochrane *Q* test statistic (*Q* test *p*=0.001) and *I*^2^ = 97.76% which was substantial heterogeneity and an indicative for using random-effects model.

**Figure 4:**
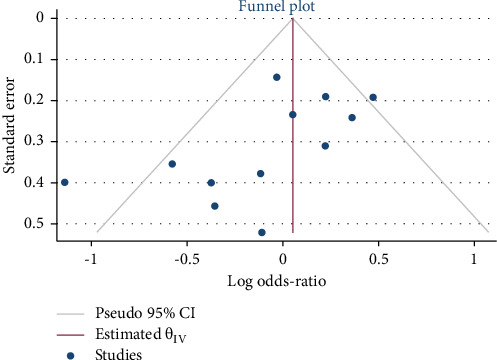
Funnel plot that assess small study effect.

The possible small study effect across the studies was observed by a funnel plot and nonparametric rank correlation (Begg) test. The funnel plot and Begg's test indicated small study effect was observed (Begg's regression tests *p* values = 0.1494) Figure 4. The symmetry of the funnel plot also indicated that there was small study effect.

### 4.5. Subgroup Analysis

**Figure 5:**
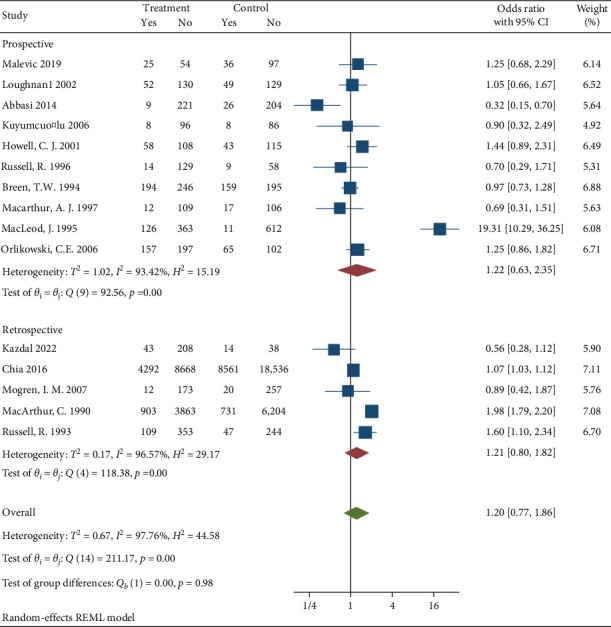
Subgroup analysis based on data collection method.

**Figure 6:**
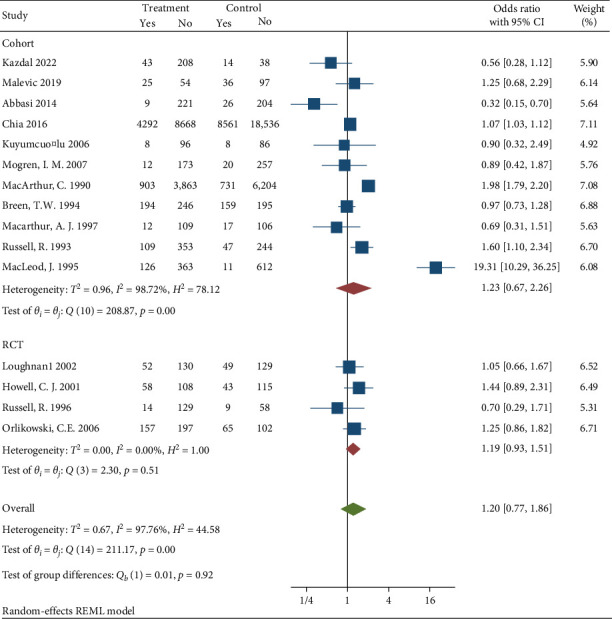
Subgroup analysis based on study design.

**Figure 7:**
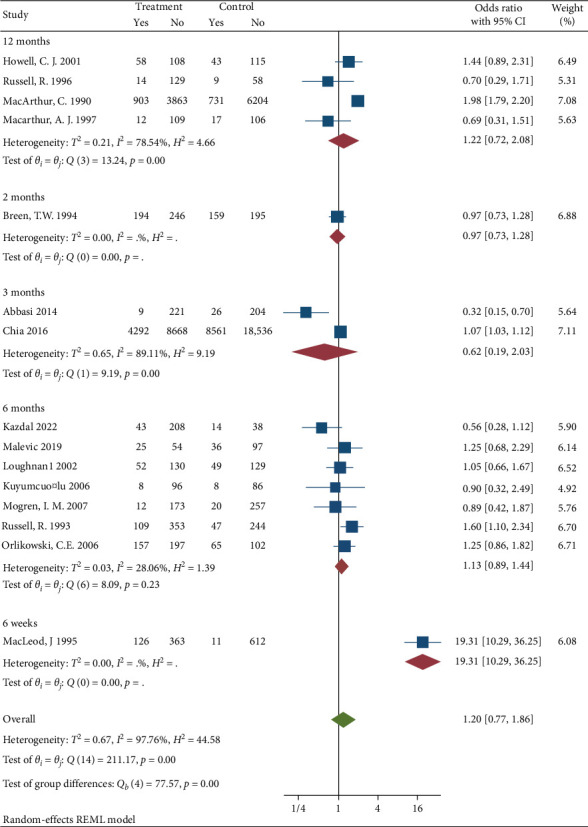
Subgroup analysis based on duration of follow up.

**Figure 8:**
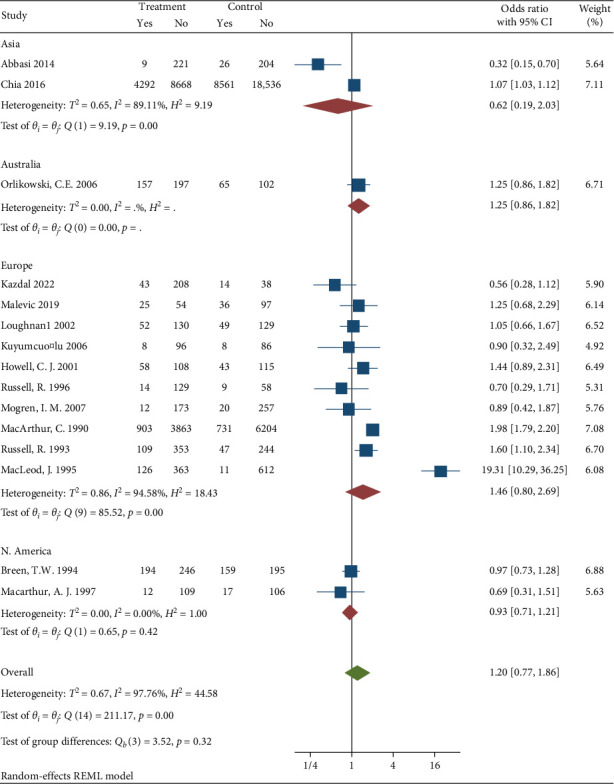
Subgroup analysis based on region in which studies were conducted.

Subgroup analysis was conducted to diagnosis the possible source of heterogeneity by data collection method Figure 5, study design Figure 6, follow up Figure 7, and region Figure 8. The subgroup analysis showed that one possible cause for heterogeneity could be study design since the studies conducted with RCT design were homogeneous with *I*^2^ = 0.00, *H*^2^ = 1, and *Q* statistics *p* value = 0.51. Duration of follow up subgroup analysis may be another source of heterogeneity since the studies that had 6 months follow up were homogeneous with *I*^2^ = 28.06, *H*^2^ = 1.39, and *Q* statistics *p* value = 0.23. The rest of the variables used for subgroup analysis were unable to identify the source of heterogeneity.

**Figure 9:**
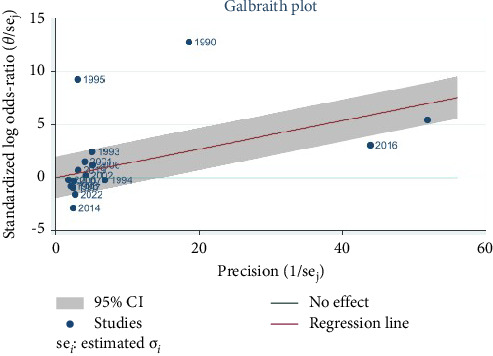
Galbraith plot to diagnose the sources of heterogeneity.

**Figure 10:**
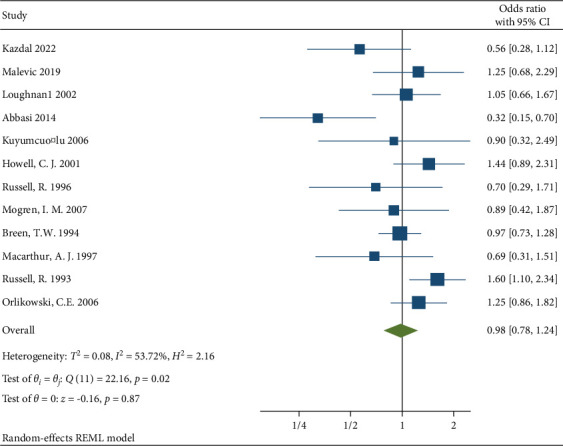
Forest plot on the associations between neuraxial anesthesia and back pain after delivery in all 12 studies.

In order to address the significant heterogeneity between studies we decided to remove three studies with a large sample sizes that were shown to be a source of heterogeneity from the Galbraith Plot Figure 9, Chia et al. (sample size = 40,957) [15] and MacArthur et al. (sample size = 11,701) [10], and MacLeod et al. (sample size = 984) [6]. This strategy resulted in a significant reduction in heterogeneity with *I*^2^ = 56.7%, *H*^2^ = 2.16, and *Q* statistics *p* value = 0.02, which was still a moderate heterogeneity. The pooled OR according to random effect Restricted maximum—likelihood model for the rest of the 12 studies was 0.98 (95% CI (0.78–1.24) *p* value = 0.87 Figure 10.

## 5. Discussion

The main clinical question addressed by this review was, the association between back pain after delivery and neuraxial anesthesia. The review included 15 studies 11 of which were cohort design, while four were RCT with moderate to high quality, reporting on 57,711 participants of whom 15,810 developed back pain.

According to the results of this meta-analysis, the pooled OR is 1.2 [95% CI (0.77–1.86)] with *p* value = 0.43; there was no statistically significant association between back pain after delivery and neuraxial anesthesia. Our meta-analysis suggests that the persistent back pain that is observed in parturient at the post-partum period is not dependent on the anesthesia provided for cesarean section or labor pain. The result of our meta-analysis are in accordance with a systematic review conducted in Chicago, USA, by Benzon, Asher, and Hartrick [34], a review conducted in Liverpool, England, by Anim-Somuah et al. [35] and a systematic review conducted in Bali, Indonesia, by Aryasa et al. [36]. It contradicts the authors MacArthur et al. [10] who stated the relationship of back pain and epidural anesthesia as a causal relationship.

The incidence of persistent back pain after delivery is thought to be associated with spinal anesthesia and epidural anesthesia. The trauma during needle insertion [37], hematoma, excessive stretching of the ligaments and prolonged lithotomy delivery postural exacerbated by muscle relaxation and immobility from neuraxial anesthesia [10] is hypothesized as the mechanism for neuraxial anesthesia induced back pain after delivery.

The causal relationship between epidural analgesia and back pain MacArthur stated was hypothesizing the mechanism as the combination of labor and anesthesia. He stated that labor position (stressed posture) exaggerated by muscle relaxation, abolition of pain, and immobility from the epidural anesthesia could be the cause for the persistent postpartum back pain seen in mothers. His hypothesis was supported by the prolonged second stage labor seen with epidural anesthesia as compared to labor without epidural anesthesia [10]. This was supported by Robin Russell, 1993, who stated the cause for postpartum back pain could be stress posture nevertheless he did not find a significant relation between back pain and epidural analgesia. In fact he postulated that the high report of back pain from epidural analgesia may be due to most women's misconception about back pain after epidural analgesia [8].

Mogren and colleagues found that elective cesarean section has higher incidence of postpartum back pain than emergency cesarean section and vaginal delivery. They hypothesized the longer immobilization period during labor and elective CS could cause damage to the joints muscles and ligaments predisposing mothers to long term back pain. They also found that epidural anesthesia and spinal anesthesia had protective effect against back pain [32]. However, it is crucial to consider the study's limitations, including a small sample size and the presence of prior unspecified back pain in 32% of participants, as well as an inadequate description of the anesthetic techniques used.

The prospective cohort study by Malevic, Jatuzis, and Paliulyte found that labor duration was longer in the epidural analgesia group, averaging an increase of 37 min [28]. Abbasi et al. also noted a link between prolonged labor and epidural use [14]. However, despite this extended duration, there was no impact on the incidence of back pain, contrary to MacArthur's assertions.

Spinal anesthesia had no significant effect on the incidence of postpartum back pain on the prospective cohort study conducted by Kazdal et al. Unilateral back pain after spinal anesthesia was seen different from other studies on epidural anesthesia, which they believed can be due to postural and structural changes during pregnancy like increased lordotic posture, weight gain, and spinal imbalance [17].

Based on the cumulative analysis the incidence of back pain associated with neuraxial anesthesia seems to have decreased over time. This might be because mothers experience less labor prolongation and greater mobility, the risk factors traditionally linked to back pain, such as prolonged immobility and stress on the back, may be mitigated. The emphasis on early mobility in postpartum care helps strengthen muscles and improve posture, potentially reducing discomfort. Therefore, these evolving practices in neuraxial anesthesia could contribute to the observed decrease in persistent back pain over time, highlighting a significant shift in how labor and recovery are managed.

Based on our findings the alternative causes of persistent back pain after delivery can be suggested. Hormonal changes during pregnancy, particularly the increase in relaxin, can lead to joint laxity and instability, predisposing women to back pain [38]. Additionally, physical changes in posture due to weight gain, increased lordosis, and shifts in the center of gravity can contribute to discomfort. The demands of labor, including pushing and prolonged positions, may strain back muscles and ligaments. Prolonged immobility during labor, especially in the lithotomy position, can result in stiffness and muscle weakness, exacerbating postdelivery pain [39]. Lastly, poor posture during breastfeeding can strain the back and lead to persistent discomfort. These factors suggest that while neuraxial anesthesia may not significantly influence postpartum back pain, various physiological and lifestyle changes associated with pregnancy and childbirth likely play a crucial role in its development. Further studies may help clarify these associations and identify effective preventive strategies.

There were limitations to our study, there was significant heterogeneity and inconsistency between studies included. We were not able to diagnose the source of heterogeneity even though we performed diagnostic analysis. There is also a small study effect seen through funnel plot and Begg's test. Another limitation is that the studies included have not adequately considered the impact of psychosocial factors such as anxiety and depression on back pain outcomes. Additionally, variations in the types and gauges of spinal and epidural needles used in procedures, as well as the assessment of post-dural puncture symptoms particularly headaches and their management through epidural blood patches have not been thoroughly examined. These factors may significantly influence the overall understanding of back pain outcomes.

## 6. Conclusion

We can conclude that neuraxial anesthesia may not be the cause or the risk factor for the overwhelmingly high incidence of back pain women experience after delivery. On the basis of our result, we recommended the use neuraxial anesthesia for labor pain and cesarean delivery as without the fear of postpartum back pain. Although we advise caution in interoperation of our data since there is a significant heterogeneity and inconsistency seen between the studies included. We believe there is a need for further study to identify the real cause of back pain in women after delivery.

## Figures and Tables

**Table 1:** Characteristics of included studies in the meta-analysis.

No.	Author	Study period	Region	Data collection	Study design	Follow up period	Previous back pain	Sample size	Groups SS	Delivery methods	Incidence of back pain %	BP after delivery	Response rate (%)	Quality score
1	Kazdal et al. [17]	2022	Turkey	Retrospective	Cohort	3 months 6 months	No	303	251 52	SA GA	17.1 26.9	43 14	100	High
2	Malevic, Jatuzis, and Paliulyte [28]	2019	Lithuania	Prospective	Cohort	6 months	No	212	79 87 46	EA IVA NA	31.65 28.74 23.91	25 25 11	73.8	Mid
3	Koughnan et al. [29]	2002	England	Prospective	RCT	6 months	No	360	182 178	EA IVA	29 28	52 49	85	Mid
4	Abbasi et al. [14]	2014	Pakistan	Prospective	Cohort	3 months	No	460	230 230	EA NA	3.9 11.3	9 26	95	High
5	Chia et al. [18]	2016	Taiwan	Retrospective	Cohort	3 months	No	40,057	4298 8662 27097	EA SA NA	35.74 31.82 31.59	1536 3756 8561	100	High
6	Kuyumcuoğlu et al. [30]	2006	Turkey	Prospective	Cohort	1 month 6 months	No	198	104 94	CES NA	7.7 8.5	8 8	100	High
7	Howell et al. [31]	2001	England	Prospective	RCT	3 month 1 year	Yes	317	166 151	EA NEA	35 27	58 43	88	High
8	Russell, Dundas, and Reynolds [12]	1996	England	Prospective	RCT	3 months 1 year	No	210	64 79 67	EA LDEA NEA	6.4 8.6 6.9	10 4 9	75	Mid
9	Mogren [32]	2007	Sweden	Retrospective	Cohort	6 months	Yes	462	185 277	EA NEA	6.5 7.2	12 20	83.2	Mid
10	MacArthur et al. [10]	1990	England	Retrospective	Cohort	1 year	No	11701	4766 6935	EA NEA	18.9 10.5	903 731	79	Mid
11	Breen et al. [9]	1994	USA	Prospective	Cohort	1 months 2 months	Yes	794	440 354	EA NEA	49 45	194 159	88	High
12	Macarthur, Macarthur, and Weeks [5]	1997	Canada	Prospective	Cohort	1 year	No	244	121 123	EA NEA	10 14	12 17	74.2	High
13	Russell et al. [8]	1993	England	Retrospective	Cohort	6 months	No	753	462 291	EA NEA	23.6 16.2	109 47	62.9	High
14	MacLeod et al. [6]	1995	Scotland	Prospective	Cohort	6 weeks	No	984	492 632	EA NEA	26 2	126 11	67.1	High
15	Orlikowski et al. [33]	2006	Australia	Prospective	RCT	2 months 6 months	Yes	992	354 167	EA NEA	22.7 21.5	157 65	52.5	Mid

Abbreviations: EA, epidural anesthesia; GA, general anesthesia; IVA, intravenous anesthetics; NA, no anesthesia; NEA, no epidural anesthesia; SA, spinal anesthesia.

## Data Availability

Data are available to be presented upon request.

## Nomenclature

ASA: American society of anesthesiologist
BMI: Body mass index
CS: Cesarean section
CSE: Combined spinal-epidural anesthesia
EA: Epidural anesthesia
IVA: Intravenous anesthesia
LBP: Lower back pain
NA: No anesthesia
NPRS: Numeric pain rating scale
PSBP: Postspinal back pain
RCT: Randomized control trial
SA: Spinal anesthesia.

## Supporting Information

Supporting Information 1 Supporting file 1: quality assessment, JBI Critical Appraisal Checklist for cohort and RCT studies.

Supporting Information 2 Supporting file 2: List of articles that underwent full article review.
