# Supplementary material for: Assessment of the Association Between Neuraxial Anesthesia and Back Pain After Delivery: A Systematic Review and Meta-Analysis
Source: Anesthesiol Res Pract. 2025 Jan 29;2025:2105413. doi: 10.1155/anrp/2105413 (PMC11824844; doi:10.1155/anrp/2105413)
Supplement: Supporting Information 1 — Supporting file 1: quality assessment, JBI Critical Appraisal Checklist for cohort and RCT studies. [file 2105413.f1.docx]

**JBI Critical Appraisal Checklist for cohort studies**

| No | Cohort JBI quality assessment | Kazdal.2022 | Malevic.2019 | Abbasi.2014 | Chia.2016 | Mogren, I. M.2007 | Kuyumcuo¤lu.2006 | MacArthur, C.1990 | Breen, T.W1994 | Macarthur, A. J.1997 | Russell, R.1993 | MacLeod, J 1995 |
| --- | --- | --- | --- | --- | --- | --- | --- | --- | --- | --- | --- | --- |
| 1 | Were the two groups similar and recruited from the same population? | No | Yes | No | No | Yes | Yes | No | No | No | No | No |
| 2 | Were the exposures measured similarly to assign people to both exposed and unexposed groups? | Yes | Yes | Yes | Yes | Yes | Yes | Yes | Yes | Yes | Yes | Yes |
| 3 | Was the exposure measured in a valid and reliable way? | Yes | Yes | Yes | Yes | Yes | Yes | Yes | Yes | Yes | Yes | Yes |
| 4 | Were confounding factors identified? | No | No | Yes | Yes | Yes | No | No | Yes | Yes | Yes | Yes |
| 5 | Were strategies to deal with confounding factors stated? | No | No | Yes | Yes | No | No | No | Yes | Yes | Yes | Yes |
| 6 | Were the groups/participants free of the outcome at the start of the study (or at the moment of exposure)? | Yes | Yes | Yes | Yes | No | Yes | Yes | No | No | Yes | Yes |
| 7 | Were the outcomes measured in a valid and reliable way? | No | No | No | No | No | No | No | No | No | No | No |
| 8 | Was the follow up time reported and sufficient to be long enough for outcomes to occur? | Yes | Yes | Yes | Yes | Yes | Yes | Yes | No | Yes | Yes | Yes |
| 9 | Was follow up complete, and if not, were the reasons to loss to follow up described and explored? | Yes | No | Yes | No | No | Yes | No | Yes | Yes | Yes | No |
| 10 | Were strategies to address incomplete follow up utilized? | Yes | No | No | No | No | Yes | Yes | Yes | No | Yes | No |
| 11 | Was appropriate statistical analysis used? | No | Yes | Yes | Yes | Yes | No | yes | Yes | Yes | No | Yes |
| 12 | Were the two groups similar and recruited from the same population? | Yes | Yes | Yes | Yes | Yes | Yes | Yes | Yes | Yes | Yes | Yes |
| 13 | Were the exposures measured similarly to assign people to both exposed and unexposed groups? | Yes | Yes | Yes | Yes | Yes | Yes | Yes | Yes | Yes | Yes | Yes |
|  | Total result | 9 | 8 | 10 | 9 | 8 | 9 | 8 | 9 | 9 | 10 | 9 |
|  | Quality | High | Mid | High | High | Mid | High | Mid | High | High | High | High |

Yes No Unclear Not applicable

Quality classification

1. High quality – 9-13
2. Medium quality – 8-7
3. Low quality <7

| No | Internal Validity | C.J. Howell, 2001 | Loughnan1.2002 | Robin Russell, 1996 | Orlikowski, C.E 2006 |
| --- | --- | --- | --- | --- | --- |
|  | **Bias related to selection and allocation** |  |  |  |  |
| 1 | Was true randomization used for assignment of participants to treatment groups? | Yes | Yes | Yes | Yes |
| 2 | Was allocation to treatment groups concealed? | Yes | Yes | No | No |
| 3 | Were treatment groups similar at the baseline? | Yes | Yes | No | Yes |
|  | **Bias related to administration of intervention/exposure** |  |  |  |  |
| 4 | Were participants blind to treatment assignment? | No | No | No | No |
| 5 | Were those delivering the treatment blind to treatment assignment? | No | No | No | No |
| 6 | Were treatment groups treated identically other than the intervention of interest? | Yes | Yes | Yes | Yes |
|  | **Bias related to assessment, detection and measurement of the outcome** |  |  |  |  |
| 7 | Were outcome assessors blind to treatment assignment? |  |  |  |  |
|  | Outcome 1 | No | No | No | No |
| 8 | Were outcomes measured in the same way for treatment groups? |  |  |  |  |
|  | Outcome 1 | Yes | Yes | Yes | Yes |
| 9 | Were outcomes measured in a reliable way |  |  |  |  |
|  | Outcome 1 | No | No | No | No |
|  | **Bias related to participant retention** |  |  |  |  |
| 10 | Was follow up complete and if not, were differences between groups in terms of their follow up adequately described and analysed? |  |  |  |  |
|  | Outcome 1 |  |  |  |  |
|  | Result | Yes | Yes | Yes | Yes |
|  | **Statistical Conclusion Validity** |  |  |  |  |
| 11 | Were participants analysed in the groups to which they were randomized? |  |  |  |  |
|  | Outcome 1 | Yes | No | Yes | Yes |
| 12 | Was appropriate statistical analysis used? |  |  |  |  |
|  | Outcome 1 | Yes | Yes | Yes | Yes |
| 13 | Was the trial design appropriate and any deviations from the standard RCT design (individual randomization, parallel groups) accounted for in the conduct and analysis of the trial? | Yes | Yes | Yes | Yes |
|  | Total result | 9 | 8 | 7 | 8 |
|  | Quality | High | Mid | Mid | Mid |

Yes No Unclear Not applicable
