# Supplementary material for: Assessment of the Association Between Neuraxial Anesthesia and Back Pain After Delivery: A Systematic Review and Meta-Analysis
Source: Anesthesiol Res Pract. 2025 Jan 29;2025:2105413. doi: 10.1155/anrp/2105413 (PMC11824844; doi:10.1155/anrp/2105413)
Supplement: Supporting Information 2 — Supporting file 2: List of articles that underwent full article review. [file 2105413.f2.docx]

List of articles that underwent full article review

| No | Studies |
| --- | --- |
| 1 | Abbasi, S., et al., Prevalence of low back pain experienced after delivery with and without epidural analgesia: A non-randomised prospective direct and telephonic survey. Indian J Anaesth, 2014. 58(2): p. 143-8. |
| 2 | Breen, T.W., et al., Factors associated with back pain after childbirth. Anesthesiology, 1994. 81(1): p. 29-34. |
| 3 | Chia, Y.-Y., et al., Risk of chronic low back pain among parturients who undergo cesarean delivery with neuraxial anesthesia: a nationwide population-based retrospective cohort study. Medicine, 2016. 95(16). |
| 4 | Howell, C.J., et al., Randomised study of long term outcome after epidural versus non-epidural analgesia during labour. BMJ, 2002. 325(7360): p. 357. |
| 5 | Kazdal, H., et al., Does the anesthesia technique of cesarean section cause persistent low back pain after delivery? A retrospective analysis. Eur Spine J, 2022. 31(12): p. 3640-3646. |
| 6 | Kuyumcuoğlu, C., et al., The relationship of combined spinal-epidural analgesia and low-back pain after vaginal delivery. Agri, 2006. 18(3): p. 24-9. |
| 7 | Loughnan, B.A., et al., Epidural analgesia and backache: a randomized controlled comparison with intramuscular meperidine for analgesia during labour. Br J Anaesth, 2002. 89(3): p. 466-72. |
| 8 | MacArthur, C., et al., Epidural anaesthesia and long term backache after childbirth. BMJ, 1990. 301(6742): p. 9-12. |
| 9 | Macarthur, A.J., C. Macarthur, and S.K. Weeks, Is epidural anesthesia in labor associated with chronic low back pain? A prospective cohort study. Anesth Analg, 1997. 85(5): p. 1066-70. |
| 10 | Malevic, A., D. Jatuzis, and V. Paliulyte, Epidural Analgesia and Back Pain after Labor. Medicina (Kaunas), 2019. 55(7). |
| 11 | Mogren, I.M., Does caesarean section negatively influence the post-partum prognosis of low back pain and pelvic pain during pregnancy? Eur Spine J, 2007. 16(1): p. 115-21 |
| 12 | Russell, R., et al., Assessing long term backache after childbirth. BMJ, 1993. 306(6888): p. 1299-303. |
| 13 | Russell, R., R. Dundas, and F. Reynolds, Long term backache after childbirth: prospective search for causative factors. BMJ, 1996. 312(7043): p. 1384- |
| 14 | MacLeod, J., et al., *Backache and epidural analgesia: a retrospective survey of mothers 1 year after childbirth.* Int J Obstet Anesth, 1995. **4**(1): p. 21-5. |
| 15 | Orlikowski, C.E., et al., *Intrapartum analgesia and its association with post-partum back pain and headache in nulliparous women.* Aust N Z J Obstet Gynaecol, 2006. **46**(5): p. 395-401. |
| 16 | Kumari, R., et al., A Randomized Comparative Study on Median and Paramedian Approaches for Subarachnoid Block using Sprotte Needle in the Cesarean Section: Quest for the Best. Anesth Essays Res, 2021. 15(3): p. 268-271. |
| 17 | Butler, R. and J. Fuller, Back pain following epidural anaesthesia in labour. Can J Anaesth, 1998. 45(8): p. 724-8. |
| 18 | Clark, V.A. and M.A. McQueen, Factors influencing backache following epidural analgesia in labour. Int J Obstet Anesth, 1993. 2(4): p. 193-6. |
| 19 | Shutt, L.E., et al., Spinal anaesthesia for caesarean section: comparison of 22-gauge and 25-gauge Whitacre needles with 26-gauge Quincke needles. Br J Anaesth, 1992. 69(6): p. 589-94. |
| 20 | Davies, S.J., et al., Maternal experience during epidural or combined spinal-epidural anesthesia for cesarean section: a prospective, randomized trial. Anesth Analg, 1997. 85(3): p. 607-13. |
| 21 | Scott, D.B. and M.E. Tunstall, Serious complications associated with epidural/spinal blockade in obstetrics: a two-year prospective study. Int J Obstet Anesth, 1995. 4(3): p. 133-9 |
| 22 | Akdemir, M.S., et al., The Postdural Puncture Headache and Back Pain: The Comparison of 26-gauge Atraucan and 26-gauge Quincke Spinal Needles in Obstetric Patients. Anesth Essays Res, 2017. 11(2): p. 458-462. |
| 23 | Aktham Adelshoukry, A.A., Randomized Control Study for Evaluation of Effectiveness of Topical NSAIDS Patches to Prevent Backache Following Spinal Anesthesia in Caesarean Section. Open Journal of Anesthesiology, 2018. 8: p. 35-42. |
